# Identification of Constrained Cancer Driver Genes Based on Mutation Timing

**DOI:** 10.1371/journal.pcbi.1004027

**Published:** 2015-01-08

**Authors:** Thomas Sakoparnig, Patrick Fried, Niko Beerenwinkel

**Affiliations:** 1 Department of Biosystems Science and Engineering, ETH Zürich, Basel, Switzerland; 2 SIB Swiss Institute of Bioinformatics, Basel, Switzerland; National Research Council of Canada, Canada

## Abstract

Cancer drivers are genomic alterations that provide cells containing them with a selective advantage over their local competitors, whereas neutral passengers do not change the somatic fitness of cells. Cancer-driving mutations are usually discriminated from passenger mutations by their higher degree of recurrence in tumor samples. However, there is increasing evidence that many additional driver mutations may exist that occur at very low frequencies among tumors. This observation has prompted alternative methods for driver detection, including finding groups of mutually exclusive mutations and incorporating prior biological knowledge about gene function or network structure. Dependencies among drivers due to epistatic interactions can also result in low mutation frequencies, but this effect has been ignored in driver detection so far. Here, we present a new computational approach for identifying genomic alterations that occur at low frequencies because they depend on other events. Unlike passengers, these constrained mutations display punctuated patterns of occurrence in time. We test this driver–passenger discrimination approach based on mutation timing in extensive simulation studies, and we apply it to cross-sectional copy number alteration (CNA) data from ovarian cancer, CNA and single-nucleotide variant (SNV) data from breast tumors and SNV data from colorectal cancer. Among the top ranked predicted drivers, we find low-frequency genes that have already been shown to be involved in carcinogenesis, as well as many new candidate drivers. The mutation timing approach is orthogonal and complementary to existing driver prediction methods. It will help identifying from cancer genome data the alterations that drive tumor progression.

## Author Summary

Cancer genome sequencing projects result in vast amounts of cancer mutation data. However, our understanding of which mutations are driving tumor growth and which are selectively neutral is lagging behind. Functional interactions among mutations can result in mutational dependencies, and these mutations then display low marginal mutation frequencies across tumor samples complicating the identification of these drivers. Here, we present a simple method for calling candidate driver mutations by discriminating dependent mutations from independent ones based on their dynamical patterns of occurrence. The gene ranking procedure measures deviation from neutral mutation timing patterns. We demonstrate, for different types of cancers and genetic alterations, improvement over classical frequency-based approaches if drivers do not occur independently, and we show complementarity to other approaches.

## Introduction

Carcinogenesis is an evolutionary process driven by the accumulation of advantageous mutations in single cells and the subsequent outgrowth of those cells due to clonal expansion. Mutations in certain genes are present in a large fraction of cancers, such as *TP53* mutations; others exhibit high mutation rates in cancers of the same type such as *BRCA1* in breast cancer. The functional alterations of these recurrently mutated genes are referred to as hallmarks of cancer [1].

However, cancer genomes contain many more mutations which do not show high degrees of recurrence. There are several reasons for these low rates of recurrence. Firstly, mutations at some loci depend on the presence of mutations at other loci [2]. This dependence may result from an epistatic interaction where a mutation is selectively advantageous only in the context of other mutations. However, cancer diagnoses do not occur at the same point during carcinogenesis. Instead, some tumors are detected very early, some very late, and most tumors are diagnosed at intermediate stages. Therefore, mutations that are highly dependent on other mutations tend to occur late, viz. after the right genetic background has evolved. Hence they are present only in a small fraction of patients. Secondly, mutations in single members of functional groups, such as signaling pathways, are often sufficient to disturb the pathway function. Mutations within those pathways display patterns of mutual exclusivity and low mutation rates across cancer samples, because additional mutations are unlikely as they do not provide an additional selective advantage [3], [4]. Finally, cancer cells accumulate a large number of passenger mutations in the process of carcinogenesis [5]. These mutations are selectively neutral and occur at random, but they are also manifested within a cancer cell population due to their co-occurrence with advantageous driver mutations. The goal of driver–passenger discrimination is to separate these harmless passenger mutations from driver mutations which actually provide a selective advantage and drive tumor growth.

Driver–passenger classification approaches fall into three categories. They are either based on (i) mutation frequencies, (ii) mutual exclusivity, or (iii) biological pathway or network information. Mutation frequency-based methods aim at finding either genome-wide or locus-specific mutation frequency cutoffs which are optimal with respect to a given false positive rate or other criterion [6]–[8]. Mutual exclusivity-based approaches try to find sets of genes in which mutations are mutually exclusive, while most of the cancer samples display a mutation in one of the genes in these sets [9]–[11]. The third class of methods relies on enrichment of the driver candidates in annotation databases or in specific subgroups of biological networks [12]. Furthermore, combination of different methods has been used to get high confidence predictions of drivers [13]. Besides computational techniques, experimental approaches are also employed in order to identify driver genes [14]. However, experimental approaches are limited to mutations which show an effect on their own and do not depend on a more complex mutational context. With the exception of mutual exclusivity-based methods, all the above methods assume that the selective advantage of drivers is independent of the mutational and signaling context in which they appear. Mutual exclusivity-based methods assume that the effect of a driver is only present in the absence of other specific mutations.

**Figure 1:**
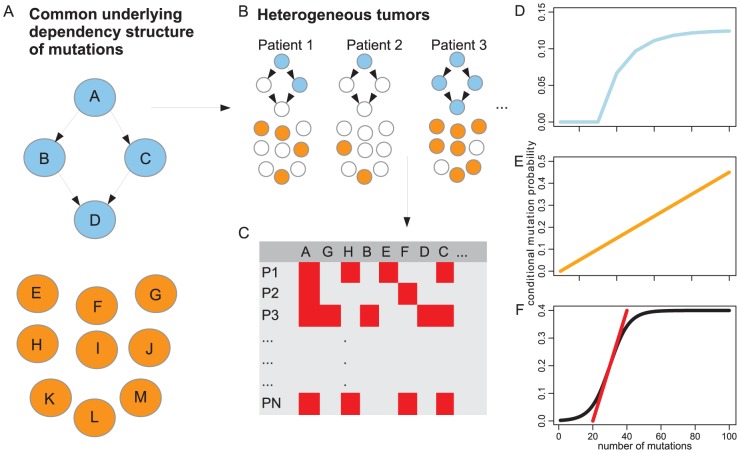
Schematic overview of the mutation accumulation process and the mutation timing approach to separate dependent from independent events. (A) The occurrence of drivers is subject to hidden constraints, represented by a dependency structure (blue), whereas passengers are independent (orange). (B) Every cancer sample is an independent realization of the common underlying oncogenesis process. (C) Noisy cross-sectional mutation data from a set of tumor samples is the basis for discrimination of dependent from independent events. (D) Conditional mutation probabilities *P*_m_(*k*)  =  Pr(mutation *m* has occurred given *k* or less mutations have occurred so far) of dependent mutations (blue) and (E) unconstrained mutations (orange) under the assumption of identical evolutionary rates have different characteristic shapes. (F) Schematic representation of a sigmoidal curve 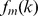 (black) used to approximate 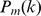 and the slope of this curve at the inflection point (red); this slope is used for ranking the genes or loci of interest.

Since some driver genes are dependent on mutations in other genes, their mutation frequency across samples can be low. Here, we describe a computational approach to identify driver genes that have low mutation frequencies due to their conditional occurrence. The method takes cross-sectional binary mutation data as input and aims to separate dependent events from independent ones. Our approach is based on the observation that the probabilities of neutral mutations increase linearly with the total number of mutations, which is used here as a proxy for the timespan between the start of oncogenesis and the detection of the tumor. By contrast, non-neutral mutations that depend on other mutations occur with probabilities displaying a non-linear pattern of punctuated increase (Fig. 1).

Extensive simulation studies are used in order to evaluate the best-case performance of the mutation timing approach and to assess its dependency on several factors, including the number of samples, the genotyping error rate, and the variation of the background mutation rates. We analyze real CNA datasets from ovarian and breast cancer as well as SNV datasets from breast and colorectal cancer obtained from the TCGA database. We find a number of known oncogenes and tumor suppressors to be highly ranked as well as genes which have not yet been implicated in tumor development. Furthermore, we find a very low overlap of genes highly ranked by our mutation timing approach and genes which have high degrees of mutation recurrence in the datasets we analyzed, indicating complementarity between our novel and existing approaches.

## Results

### Timing of independent versus dependent mutations

In order to identify candidate cancer driver genes, we propose to detect mutations whose occurrence depends on the presence of other mutations (Fig. 1). The rationale for this approach is that passenger mutations occur independently because they are selectively neutral, whereas the selective advantage of many driver mutations depends on the genetic background they occur in. For example, mutation of *KRAS* tends to occur after mutation or loss of *FAP* in colorectal tumorigenesis [2]. To distinguish independent from dependent mutations, we study their rate of occurrence among tumors. Independent mutations occur at a constant rate (Fig. 1E), and our driver–passenger discrimination approach is based on detecting deviation from this behavior. For example, if mutations occur in a linear order, 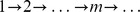, then mutation 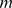 can occur only after all its predecessors have occurred, and once this has happened, the probability of mutation 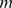 increases much faster than in the neutral case (Fig. 1D; S1 Text, section 3.2). This difference in the rate of change of observing mutations over time is the basis for our gene ranking procedure.

For each mutation *m*, we consider the conditional probability of its occurrence given that in total at most 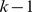 mutations have accumulated,
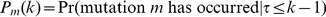
(1)


Here 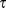 is the total number of mutations, which we use as a proxy for the time of observation relative to the onset of tumorigenesis. Thus, we measure time in number of mutations, 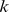, and 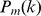 is the probability of observing mutation 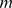 before time 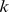. For independent mutations occurring at identical rates, 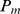 is a linear function with constant slope 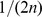, where 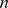 is the total number of possible mutations (Fig. 1E; S1 Text, Eq. 21). By contrast, for dependent mutations, 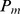 is non-linear with a sharp increase in a more confined time interval. Furthermore, we demonstrated that if 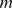 is involved in dependency relations, then 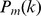 has a higher maximal slope than for independent mutations (S1 Text, Theorem 1).

**Figure 2:**
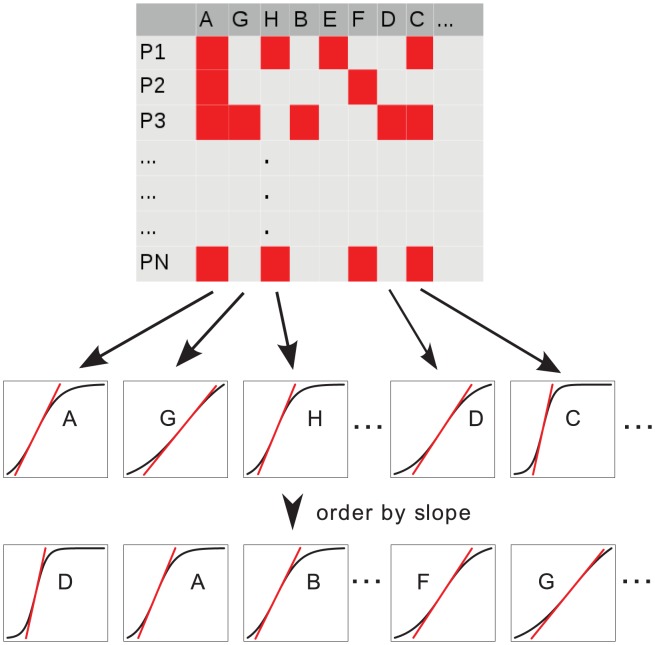
Mutation timing schema. Sigmoidal curves are fitted for every gene and genes are subsequently ranked according to their likelihood of having constraints, i.e., the steepest slope of the sigmoidal curve.

This finding shows that mutational dependencies can be detected by considering the steepest slope of 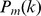. We model the probability 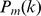 using the sigmoid function 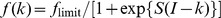, where 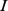 is the location of the inflection point, 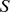 the slope at 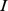, and 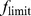 the mutation frequency over all samples (Fig. 1F; S1 Text, section 4.1). Mutation timing ranking then ranks genes by decreasing slope values, 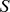. Genes that tend to have the same probability of occurrence over time will be ranked low, whereas genes with a narrow window of high occurrence probability are ranked high (Fig. 2).

### Simulation studies

In order to evaluate the performance of the mutation timing ranking, we conducted simulation studies and compared our approach to the baseline frequency-based approach. We ranked the simulated genes by the mutation timing method and by their marginal frequency and computed and compared AUC values for both rankings. We simulated samples according to a continuous-time Conjunctive Bayesian Network model [15], in which dependencies among binary mutational events are represented by a directed acyclic graph (Fig. 1A). After generating the mutation profiles we added noise by flipping every mutation indicator with probability 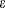 (Methods).

The probabilities 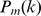 of all mutations that are early to intermediate in the dependency structure or are independent of the other nodes can be described very well by the sigmoidal approximation (S1 Fig.). Late mutations with low marginal frequencies suffer from higher fluctuations due to small sample sizes. They are affected more by measurement noise (S1 Fig.).

**Figure 3:**
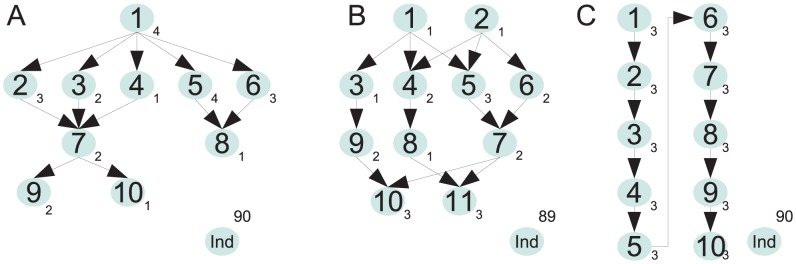
The three mutation dependency networks used for simulation. Nodes represent genes (or other mutatable entities, for example, pathways) and arrows represent mutational dependencies. Evolutionary rate parameters (parameterizing exponential waiting time distributions for modeling the time until an event happens) for each mutation are given next to the respective node. The evolutionary rate parameters for the independent nodes were drawn from various distributions in the different simulation settings. The mutation processes are stopped at the time of observation after an exponentially distributed waiting time with rate parameter 1 for all simulations. (A) Dependency network used for simulation with ten dependent nodes. (B) Dependency network used for simulation with 11 dependent nodes. (C) Linear dependency network with ten dependent nodes.

**Figure 4:**
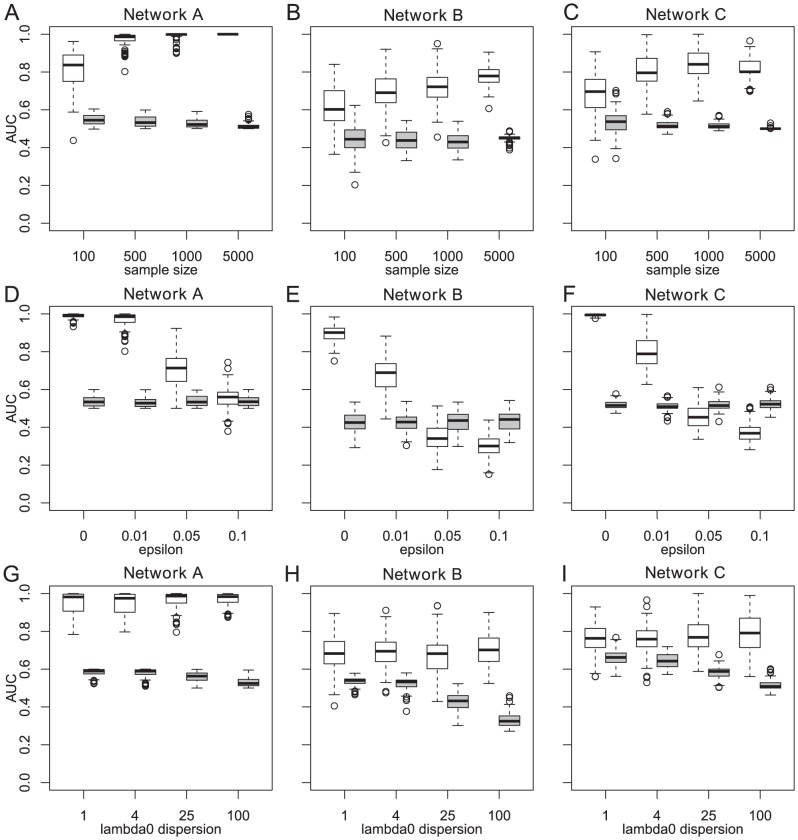
Performance of mutation timing and frequency-based gene ranking in simulation studies on three fixed networks. The area under the ROC curve (AUC) values shown were computed from 100 simulations for each setting. The AUCs for mutation timing ranking and frequency-based ranking are always shown next to each other, with the left one (white) being the mutation timing classifier and the right one (grey) being the frequency-based classifier. (A,B and C) For networks A, B and C, respectively, the sample size was varied, and the error rate was set to 0.01. (D, E, and F) For networks A, B and C, respectively, the error rate was varied, and the sample size was set to 500. (G, H and I) For networks A, B anc C, respectively, the variation of the passenger rates, 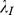, (corresponding to the independent nodes) was varied, while the error rate was constant at 0.01, and the sample size was 500.

In order to evaluate the influence of the sample size on the classifier performance, we drew samples of different sizes from three different dependency networks (Fig. 3). For the error rate, 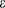, we used a value of 0.01. We applied our classifier to sets of size 100, 500, 1000, and 5000. Increasing the sample size improved the performance of our classifier (Fig. 4A–C), whereas no performance increase was observed for the frequency-based classifier.

Without observation error (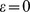) and a sample size of 500 the classifier works almost perfectly, for all three networks. With increasing error rate, 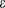, from 0 to 0.1 and sample size of 500, the performance of the classifier drops to the level of the frequency-based classifier and below in case of the linear network C (Fig. 4D–F). The performance of the frequency-based classifier is not affected by the error rate. The marginal probabilities of mutations in network B and the marginal mutation probabilities of late events of network C are lower than for network A. Therefore, ranking by mutation timing is much more sensitive to the error rate. In networks B and C, the marginal frequencies of nodes low in the dependency structure are in fact smaller than the error probability in some cases. In these cases, the mutation timing method performs worse than random. This effec is due to the sigmoidal curves getting flattened out by the noise, because the noise is uniform with rising k. The effect of the noise, i.e., the flattening of the mutation timing curve, is stronger on these low nodes than on the independent nodes because the marginal frequency of the low nodes in the dependency structure is on average smaller than the marginal frequency of the independent nodes.

Next, we investigate the influence of the variation of the waiting times of the independent nodes, i.e., the per-gene evolutionary rates. We varied the ratio between the minimum and maximum of the support of the log-uniform distribution which is used to sample the waiting time parameter for the independent nodes. The geometric mean of the minimum and maximum was kept at 0.6, 0.25, and 0.15, respectively. The ratio between the maximum and the minimum of the log-uniform distribution was set to 1, 4, 25, and 100. Then, 500 samples were drawn from the networks with an error rate of 0.01. The performance of the mutation timing classifiers on the datasets of all three networks did not change when varying the variation of the rate parameter of the independent nodes (Fig. 4G–I). The performance of the frequency-based classifier drops due to the increased number of passengers with higher marginal frequency after increasing the variability of the passenger mutation rate. Increasing the number of independent nodes (to 990, 989, and 990, respectively) hardly changed the performance of both the mutation timing and the frequency-based classifier (S2 Fig.).

**Figure 5:**
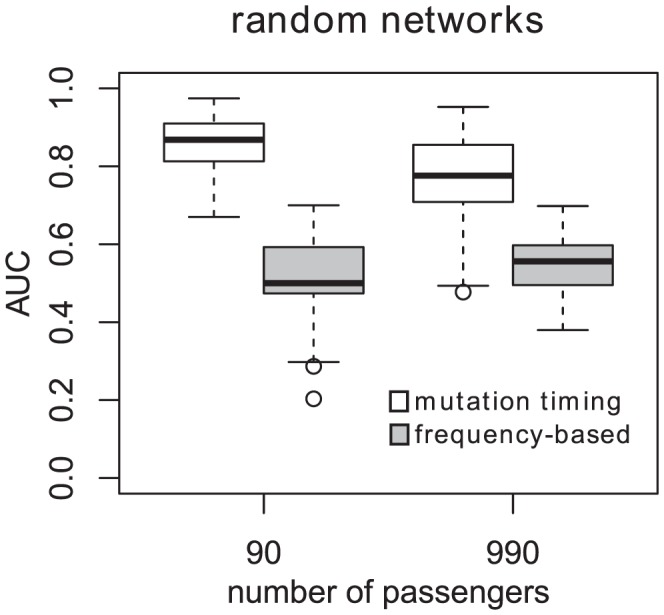
Performance of mutation timing and frequency-based gene ranking in simulation studies on random networks. Area under the ROC curve (AUC) values were computed from 100 different random networks. The AUCs for mutation timing ranking and frequency-based ranking are always shown next to each other, with the left one (white) being the mutation timing classifier and the right one (grey) being the frequency-based classifier. The error rate was constant at 0.01, and the sample size was 500.

The mutation timing classifier shows similar performance improvements over the frequency-based classifier on random networks with 10 connected nodes and 90, respectively 990 unconnected nodes (Fig. 5). The samples size for this analysis was 500 and the observation error was 0.01.

### CNAs in ovarian cancer

We applied mutation timing ranking to CNAs identified in 569 ovarian cancer samples available from the TCGA database (download on May 24, 2013). All analyzed TCGA data in this paper was downloaded via the cBio portal [16]. The CNAs were called jointly for all samples with GISTIC2 and subsequently mapped to genes for the individual tumors [17]. Only amplifications and homozygous deletions were considered. Gains and heterozygous deletions were not considered as CNAs since they are very difficult to detect and prone to high false positives. Out of 9312 CNAs, 80% were present in between 9 and 60 tumors (10% in less and 10% in more). Most tumors had between 55 and 1075 CNAs (10% less and 10% more).

**Figure 6:**
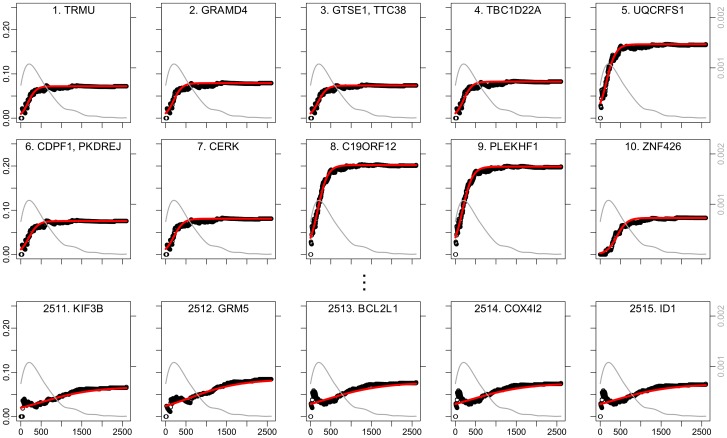
Top 10 (top two rows) and bottom 5 (bottom row) ovarian cancer copy number altered genes according to mutation timing ranking. The mutation probability 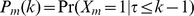 is plotted against *k*, the cumulative number of CNAs. The sigmoidal approximation 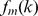 is shown in red. The grey line represents the kernel density estimate of the number of samples with *k* mutations in the study. The scale of this density is shown in grey on the right side axis of the plots.

For a certain CNA to be considered for ranking in this analysis, it had to be present in at least 35 of the 569 samples (i.e., a marginal frequency of at least 6%). Some CNAs affect multiple genes. Those genes were grouped and ranked together. The number of CNAs (genes or groups of genes), which were present in at least 35 samples, was 2515 and their sample-wise mutation profiles were unique among all 569 samples. For approximating time (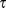), we used the overall number of CNAs present in each sample. Lowly ranked CNA-altered genes exhibit a shape which resembles a linear function as expected for passengers (Fig. 6, bottom row), while highly ranked genes exhibit a punctuated rise of their mutation frequency indicating deviation from neutrality (Fig. 6, top two rows).

Since we do not consider amplifications and losses separately in this analysis, we subsequently analyzed the type of all highly ranked CNAs. All but *ZNF426* (rank 10) show clear consistency in their type of alteration, i.e., they were either mostly deleted or mostly amplified, but not deleted in some samples and amplified in others (S3 Fig.).

Among the top-10 ranked genes (and gene groups) out of 2515 (Fig. 6, S1 Table) we find *GRAMD4* (rank 2) which has p53-like function and mediates p73-triggered apoptosis [18]. CNAs affecting *GRAMD4* are mostly homozygous or heterozygous deletions (S3 Fig.). Furthermore, amplification of *UQCRFS1* (rank 5) has been associated with high grade breast cancer [19] and enhanced colony formation in cell lines [20]. *C19ORF12* (rank 8) and *PLEKHF1* (rank 9) are almost always amplified together. Both genes were found to be essential for cell proliferation [20]. There was no overlap between the 10 most frequent CNAs and our top 10 ranked CNAs, and we found only two overlapping genes between the two top-100 lists, namely *ADAMTS10* and the *ADIPOR1*/*KLHL12* group.

### CNAs in breast cancer

Next, we applied our ranking scheme to CNA data from 913 breast cancer samples from the TCGA database (download on May 24, 2013). CNAs were called and processed in the same way as describe above for ovarian cancer. The percentage of all 8309 CNAs present in between 7 and 85 tumors (10% in less and 10% in more) was 80. Most tumors had between 4 and 622 CNAs (20% less and 10% more). Due to the higher sample number and hence increased power, we considered CNAs which are present at lower marginal frequencies than in the ovarian study. The threshold for consideration was set to 4%, or 37 of the 913 samples. This threshold was passed by 1713 CNAs, which were subsequently ranked.

Highly ranked CNAs as well as lowly ranked CNAs follow the sigmoidal shape (with different slopes) very well (S4 Fig., S2 Table). Interestingly, all top-10 ranked genes showed amplifications (S5 Fig.). The highest ranked gene is *GAL*. The Galanin signaling cascade has been proposed as a candidate pathway regulating oncogenesis in human squamous cell carcinoma [21]. *MRPL21* (rank 6), which is located near GAL in locus 11q13.2, has been suggested to play a role in carcinogenesis [21]. Similarly, *SLC29A2* (rank 4) has been implicated in the carcinogenesis of hepatocellular carcinoma [22]. *CPT1A* (rank 3) can also be found in the list of highly ranked genes. It promotes cell motility and is therefore thought to increase the risk of metastases [23].

*POLD4* (rank 8) has been associated with genomic instability in lung cancer [24]. However, the cancer driving effect of *POLD4* was associated with downregulation of this gene [24] and here we find it consistently amplified. Similarly, low levels of *KDM2A* (rank 9), a JmjC-domain containing histone demethylase, have been associated with carcinogenesis, and we find it consistently amplified [25].

### SNVs in breast cancer

Besides by CNAs, breast cancer progression is also driven by SNVs [26]. Therefore, we applied mutation timing ranking to SNV data from 772 breast cancer samples from the TCGA database (download on May 24, 2013). Since SNVs do not exhibit as high marginal frequencies as CNAs, we set the cutoff for SNVs to be ranked to 1% or 8 out of 772. The final number of ranked genes was 256.

SNVs do not show the punctuated rise in mutation frequency as pronounced as CNAs (S6 Fig.). Some of the genes display much higher conditional frequencies than marginal frequencies in early time intervals. *AOAH* and *BRCA2* are examples of this behavior (S6 Fig.). This effect could either be due to higher relative sampling fluctuations for SNVs, or it may have a biological reason. For example, tumors containing these mutations could be more aggressive and are therefore diagnosed earlier and do not have the time to accumulate more mutations.

The set of top-30 ranked genes contains a number of known drivers (S6 Fig., S3 Table). *AKT1* has been associated to breast, colorectal, and ovarian cancer formation [27]. Germline mutations of *BRCA2* are associated with increased risk for developing breast and ovarian cancer [28]. However, also somatic mutations of *BRCA2*, which are considered here, have been shown to drive cancer progression [4]. Furthermore, known drivers such as *CDH1*, *CTCF*, and *GATA3* are found within the top-30 ranked genes [26]. Of the top-30 ranked genes 16 have q-values below 0.2 (Bejamini-Hochberg). Of the rest only two more display q-values below 0.2 (S3 Table).

### SNVs in colorectal cancer

Dependencies among mutations were initially studied in colorectal cancers and adenomas [2]. However, the number of sequenced colorectal cancer samples in TCGA is still relatively small. Furthermore, it was shown that in colorectal cancer (among others) the number of mutations is correlated with the age of the patient and is therefore a poor measure of tumor age [29]. Nevertheless, we applied mutation timing ranking to SNV data from 223 colorectal cancer samples from the TCGA database (downloaded on July 31, 2014). We set the cutoff for SNVs to be ranked to 5% or 12 out of 223. Furthermore, we excluded all samples which supposedly exhibited a mutator phenotype. Therefore, we used a cut-off of 1000 SNVs in the analyzed samples. This resulted in 212 samples used for mutation timing ranking. The final number of ranked genes was 69 (S7 Fig., S4 Table). The classical colorectal cancer genes *APC*, *KRAS* and *TP53* (all rank 1) are among the top-predicted constrained genes [30]. Furthermore, known drivers *NRAS* and *PIK3CA* (rank 16 and 18, respectively) are highly ranked [31], [32]. Since the number of samples and ranked genes is very small the mutation timing-ranked list is probably unstable. Of the highly-ranked genes in colorectal cancer, *APC*, *KRAS*, and *TP53* are significant at a false discovery rate of 0.2 (Benjamini-Hochberg).

### Dependencies among highly-ranked drivers in breast cancer

In order to check for dependencies among highly-ranked cancer drivers we used the oncogenetic tree model, because it is an alternative method to our conjunctive Bayesian network model used here to motivate the mutation timing method [33]. The driver list we used is a union of different driver lists from http://www.bushmanlab.org/links/genelists accessed on August 5, 2014. In the ranked 256 breast cancer SNV-affected gene list, 62 drivers are present (S5 Table). We split the 62 identified drivers into top and bottom half according to their rank in the mutation timing ranking, i.e., 31 top drivers versus 31 bottom drivers. Subsequently, we learned oncogenetic trees separately for both (top and bottom half) subsets. Since for oncogenetic trees, there is no posterior probability for single edges available and they are very unstable, we bootstrapped the data and relearned the trees 1000 times. We considered not only direct but also indirect dependencies, i.e., the transitive closure of the trees in this analysis. If we compare dependencies with bootstrap confidence scores above 60% then the number of drivers with at least one dependency in highly-ranked genes versus lowly-ranked genes is 26 versus 2 (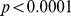, Fisher's exact test). Thus, there is an enrichment of dependent drivers in the list of highly-ranked drivers. Since CNA driver lists are very sparse we could not perform the same analysis for the mutation timing-ranked CNA genes. Furthermore, the list of ranked genes in colorectal cancer was too short for this type of analysis.

## Discussion

We have presented a driver–passenger discrimination method which specifically aims at identifying cancer-driving mutations that are constrained in their occurrence during carcinogenesis. It is known that the selective advantage of several drivers depends on the genetic background they occur in. Hence, genetic constraints that result from epistatic gene interactions have been our main motivation. However, the mutation timing approach does not make any assumption about the biological origin of the constraints. They may be unobserved and can include, for example, epigenetic, transcriptional, post-transcriptional, or environmental constraints.

The novelty of the mutation timing approach presented here is that it identifies constrained gene mutations. Several computational methods have been developed for estimating the actual dependency structure among a small set of genes [34]–[37]. Mutation timing ranking can be used to identify these genes that are amenable to modeling cancer progression in more detail. We emphasize that our mutation timing approach does not require estimating the dependency structure, which is a statistically and computationally challenging problem. Instead, we are only detecting deviation from independence of mutations.

The computational time complexity of mutation timing depends linearly on the number of genes and samples. Thus, the method can be applied efficiently to datasets of virtually any size, including genome-wide measurements of large patient cohorts. Here, we have applied mutation timing ranking to two different CNA datasets and two SNV datasets on a gene level. Once there are larger datasets available, the method can readily be applied to mutations on an amino acid or nucleotide level.

In some cancers about half of the somatic mutations found in tumor samples are assumed to be present already before tumor initiation [29]. In these cases the implicit assumption made here, that mutation accumulation starts about the same time as tumor initiation and the bulk of the mutations occurres during carcinogenesis is violated. Therefore, the results of the mutation timing method might be less stable in those cases.

We have developed a method for assessing the significance of the mutation timing-ranked genes based on a weighted permutation test. However, we only applied this test to SNV datasets here. CNAs on the gene level are highly dependent on each other because in some cancers a single CNA might be larger than in others and alter more neighboring genes then in others. It is assumed that only a small number of genes in CNA-altered regions has cancer-driving effects. However, it is very difficult to discriminate which ones are drivers and which ones are passengers. Mutation timing highly-ranked genes have often very similar CNA profiles across tumors. And significance analysis is with our test not possible because it assumes that CNAs are only dependent on timing. Therefore, we get very high numbers of significant genes. In order to make the method more appropriate for CNAs, a reliable method for counting copy number events per tumor is needed.

Mutation timing-based driver–passenger discrimination is complementary to existing approaches, including those based on mutation frequencies, mutual exclusivity, and prior network information. Thus, it can also be expected to improve the performance of ensemble methods, which integrate different classifiers [38]. Among the existing approaches, only the frequency cutoff-based approach scales to larger datasets the way mutation timing does, and our simulation studies have highlighted the improvements of mutation timing in the presence of dependencies.

Like any other approach, mutation timing has some limitations. One limitation is its difficulty to detect drivers that are already present at very early (measured by the total number of observed mutations) carcinogenesis stages, because generally there will be few samples observed at this time and most of them will already harbor such a mutation. Therefore, we can not observe a steep rise in conditional mutation frequency when increasing the number of mutations we condition on. An example of this behavior is *PTEN* in the breast cancer CNA analysis. Such a pattern may result, for example, from genetic subgroup structures among tumor samples, where a mutation is an early unconstrained event in one subgroup, but not in the others. However, these very early mutations can easily be identified by their high overall frequency in the dataset, i.e., by the complementary frequency-based approach.

Similarly, unconstrained drivers, i.e., mutations that increase the fitness of cells independently of the mutational context, can not be detected by mutation timing. They are expected to have a higher rate of occurrence than neutral mutations, but not a different temporal pattern. In general, unconstrained drivers with high mutation frequencies can again be detected by their increased frequencies. An implicit assumption made in the mutation timing as well as the frequency-based method is that changes in evolutionary rates over time do not affect the underlying driver–passenger dependency structures; they only cause the observation time point (relative to the number of observed mutations) to be shifted.

The number of available tumor samples and the accuracy of mutation calling are two additional limiting factors. Our simulation studies show that the mutation timing method performs well for datasets with more than 500 samples and genotyping error rates below 1%. Furthermore, as a rule of thumb, marginal frequencies of mutations should at least be twice as high as the average genotyping error rate. While these estimates are based on simulations under simplified conditions, they indicate that with current sample sizes and genotyping error rates, even rare driver mutations can be detected if their occurrences are constraint.

Both the CNA and SNV data analyzed here contained a small number of samples with very high numbers of CNAs and SNVs, respectively. This phenomenon is usually referred to as a mutator phenotype. The models used for simulating data in the simulation study do not capture this phenomenon and therefore only represent samples without a mutator phenotype. However, the influence of the samples with the mutator phenotype on the mutation timing method is minor, because the sigmoidal curve fits were weighted by the number of samples exhibiting a specific number of mutations.

Most driver–passenger discrimination approaches [6]–[8] assume either constant per-gene or per-base background evolutionary rates and predict as drivers those genes that exhibit significantly higher mutation frequencies in mid to large scale studies. However, these approaches ignore that different evolutionary rates could also be correlated with, for example, epigenomic context [39] and might not be due to a cancer-driving character of the affected gene. Our mutation timing method is invariant to different evolutionary rates, because the slopes we use for ranking reflect only how fast the mutation probability rises to its marginal mutation probability and not how large this marginal mutation probability is. Furthermore, epistatic interactions between cancer driving genes can cause low mutation frequencies and complicate the identification of those drivers. Our mutation timing-based cancer driver ranking approach identifies constrained candidate drivers in a computationally efficient manner. This approach will help in analyzing the upcoming data from large tumor sequencing studies which will be available in the near future.

## Methods

We have analyzed the waiting times of somatic cancer-related events in the presence and absence of order constraints using conjunctive Bayesian network models. Based on the differences we found, we have developed a method for ranking genes according to their likelihood of being contrained and hence being potential drivers. The waiting time analysis and the ranking method are described in detail in the S1 Text.

For validation, we used three different mutation networks, denoted as network A, network B, and network C to simulate datasets (Fig. 3). We sampled from a continuous-time Conjunctive Bayesian network [15], in which mutations are constrained by the occurrence of their predecessors in the network. Network A was designed such that the marginal probabilities of observing the mutations are between 0.1 and 0.8. For network B, we aimed at lower mutation probabilities and set the mutation rate parameters such that all marginal mutation probabilities were between 0.05 and 0.5. Network B had already been used for a simulation study in [35] in order to evaluate an inference method. Network C represents a linear dependency structure with marginal mutation probabilities between 0.05 and 0.8. The sampling time parameter (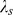), which is used to stop the time-dependent mutation accumulation process, was set to 1 for all simulation setups.

We added data from 90, 89, and 90 independent nodes, respectively, such that each network consisted of a total of 100 mutations. The mutation rate parameters for the independent nodes, denoted as 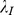, were sampled from a log-uniform distribution as follows: For fixed 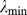 and 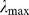, we sampled 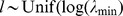, 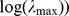 and set 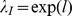. We subsequently compromised the datasets by flipping every mutation indicator with a probability of 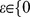, 0.01, 0.05, 0.1}.

We fitted sigmoid functions weighted by a kernel density estimator of the empirical sample distribution and ranked the features according to the slope of the fitted sigmoidal curves (Figs. 6, S4 and S6, grey lines). Most of the samples had an intermediate number of mutations and therefore contributed considerably to estimating the slope parameter, *S*. The inflection point was forced to be between 1 and the maximum number of mutations observed in the samples. The sigmoid functions were fitted by weighted least-square using the nls function from the stats package of the statistics software R [40].

In order to compute ROC curves and area under the ROC curve (AUC) values, the cutoff value for the classification was increased continuously. This procedure was repeated 100 times in order to obtain an estimate of the variability. In each iteration, the mutation rate parameters of the independent nodes, 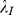, were redrawn, the underlying network and its parameters remained constant. The 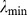 and 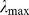 values were set to 0.06 and 6 for network A, 0.05 and 1.25 for network B and 0.015 and 1.5 for network C.

In order to generate random networks we used the following procedure: (1) Start with a set of (ten) unconnected nodes denoted as *D*. (2) Select a random unconnected node *a* from *D*. (3) Select a second random (connected or unconnected) node *b* from *D* (4) Decide if *a* becomes the parent or child node of *b* with probability 0.5. (5) Repeat from (2) until all nodes from *D* have a least one connection. This procedure ensures that all networks are valid posets, i.e, directed acyclic graphs. Six of the ten nodes had the mutation rate parameter set to 2, the rest to 1. The values of 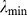 and 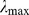 were set to 0.06 and 6 for the random networks.

For the significance analysis of the mutation timing curves, we developed a weighted permutation-based test. The null hypothesis assumes that the probability of having a mutation in a certain gene in a certain sample depends only on the marginal frequency of the mutation of this gene and on the number of mutated genes in this sample. For every gene, a specific null hypothesis is constructed. This null hypothesis keeps the marginal mutation frequency of the gene constant, i.e., the number of samples, in which it is mutated, is kept constant. Under the null hypothesis, the probability of observing a set of tumors harboring a specific mutation follows a conditional multinomial distribution with parameters proportional to the total number of mutated genes in the tumor samples and the observed number of times the gene was mutated. In each tumor, each gene can be mutated at most once, i.e., the null distribution is the multinomial conditioned on pairwise mutually exclusive events (tumors). After constructing the null distribution of the mutated genes, we fit a sigmoidal curve analogous to the other genes and record the slope. The whole procedure is repeated 1000 times. The fraction of times the slope of the randomized column was smaller than the slope of gene *i* is the p-value of gene *i*.

The R code for mutation timing-based gene ranking as well as the CNV and SNV cancer data analyzed here are available at http://www.cbg.ethz.ch/software/mutationtiming.

## Supporting Information

S1 Fig
**Examples of simulated datasets for all three networks.** Sigmoidal fits (red lines) and simulated mutation profiles for one iteration for network A, network B, and network C used in the simulation study. Top two rows from network A, middle two rows for network B, and bottom two rows from network C. Error rate (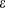) was set to 0.01 and 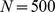 samples were drawn and used for fitting the sigmoidal curve. Mutation rate parameters for the independent nodes (passengers) were drawn from a log-uniform distribution with 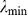 and 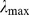 set to 0.06 and 6 for network A, to 0.05 and 1.25 for network B, and to 0.015 and 1.5 for network C. Mutation frequencies labeled “random passengers” are examples from a total of 90, 89, and 90 independent nodes.(EPS)

S2 Fig **Performance of mutation timing and frequency-based ranking in simulation studies for a total of 1000 nodes.** AUC values for various simulation settings with 990, 989, and 990 independent nodes for network A, B, and C, respectively. The AUCs shown were collected from 100 simulations, for each setting. The AUCs for the mutation timing-based ranking and the mutation frequency-based ranking are always shown next to each other, with the left box (white) describing the performance of mutation timing-based ranking. In the top row, sample size was varied. In the middle row, the error rate was varied, and in the bottom row, the variation of the independent nodes was varied.
(EPS)

S3 Fig **Distribution of copy number statuses for ovarian cancer.** Shown are histograms of the CNA status of each top 10 ovarian cancer CNA-affected gene (top two rows) and the CNA status of each of the 5 lowest ranked CNA-affected genes (bottom row). The CNA status is coded as follows: -2, homozygous deletion; -1, heterozygous deletion; 0, normal copy number; 1, gain; 2, amplification (as called by GISTIC2 [17]).
(EPS)

S4 Fig
**Top 10 (top two rows) and bottom 5 (bottom row) breast cancer copy number altered genes according to mutation timing ranking.** The mutation probability 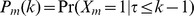 is plotted against *k*, the cumulative number of CNAs. The sigmoidal approximation 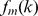 is shown in red. The grey line represents the kernel density estimate of the number of samples with *k* mutations in the study. The scale of this density is shown in grey on the right side axis of the plots.(EPS)

S5 Fig **Distribution of copy number statuses for breast cancer.** Shown are histograms of the CNA status of each top 10 breast cancer CNA-affected gene (top two rows) and the CNA status of each of the 5 lowest ranked CNA-affected genes (bottom row). The CNA status is coded as follows: -2, homozygous deletion; -1, heterozygous deletion; 0, normal copy number; 1, gain; 2, amplification (as called by GISTIC2 [17]).
(EPS)

S6 Fig **Top 30 mutation timing-ranked breast cancer SNV-affected genes (top four rows) and fits of 5 lowest ranked SNV-affected genes (bottom row).** The top-29 genes have the same rank, because the fitted slope parameters, *S*, are all equal to the upper limit of the allowed range of *S*. The sigmoid functions are shown in red. The sample density which is used for weighting the fit is shown in grey.
(EPS)

S7 Fig **Top 30 mutation timing-ranked colorectal cancer SNV-affected genes (top four rows) and fits of 5 lowest ranked SNV-affected genes (bottom row).** The top-11 genes have the same rank, because the fitted slope parameters, *S*, are all equal to the upper limit of the allowed range of *S*. The sigmoid functions are shown in red. The sample density which is used for weighting the fit is shown in grey.
(EPS)

S1 Table **Top CNAs in ovarian cancer**. Top 100 ovarian cancer copy number altered genes according to mutation timing ranking.
(CSV)

S2 Table **Top CNAs in breast cancer**. Top 100 breast cancer copy number altered genes according to mutation timing ranking.
(CSV)

S3 Table **Top SNVs in breast cancer**. Breast SNV-affected genes ranked according to mutation timing and their p-values and q-values.
(CSV)

S4 Table **Top SNVs in colorectal cancer**. Colorectal SNV-affected genes ranked according to mutation timing and their p-values and q-values.
(CSV)

S5 Table **Drivers in breast cancer SNV genes**. Driver ranks in breast cancer SNV-affected genes according to mutation timing ranking.
(CSV)

S1 Text **Accumulation dynamics of mutations based on Conjunctive Bayesian networks are studied analytically and the approximation by the mutation timing method is motivated.**
(PDF)
