# Supplementary material for: Identification of Constrained Cancer Driver Genes Based on Mutation Timing
Source: PLoS Comput Biol. 2015 Jan 8;11(1):e1004027. doi: 10.1371/journal.pcbi.1004027 (PMC4287396; doi:10.1371/journal.pcbi.1004027)
Supplement: S1 Text — Accumulation dynamics of mutations based on Conjunctive Bayesian networks are studied analytically and the approximation by the mutation timing method is motivated. (PDF) [file pcbi.1004027.s013.pdf]

Supplementary Information for "Identification of constrained cancer driver genes based on mutation timing"

Thomas Sakoparnig, Patrick Fried, and Niko Beerenwinkel

1 CBN model

The cancer-driving effect of mutations can depend on the mutation of other genes or more generally on the mutational, transcriptional, or environmental context. Epistatic interactions (among genes or between genes and other factors) introduce statistical dependencies and they can result in order constraints on the mutation accumulation during tumorigenesis. In order to study the dynamics of these order-constrained mutations and to motivate our mutation timing approach, we will consider Conjunctive Bayesian networks (CBNs), which model dependencies in binary mutation data. The continuous-time CBN (CT-CBN) model is defined by a partial order, $\prec$, on a set of n mutations and parameters λ_j, for all $j \in [n] = \{1, \ldots, n\}$. The distribution of the waiting time T_j of mutation $j \in [n]$ is defined recursively as

$$T_j \sim \mathrm{Exp}(\lambda_j) + \max_{\ell \in \mathrm{pa}(j)} T_\ell \tag{1}$$

where $\mathrm{Exp}(\lambda_j)$ denotes the exponential distribution and $\mathrm{pa}(j) = \mathrm{pa}_\prec(j)$ denotes the parents of j in the partially ordered set (poset) $([n], \prec)$. The parents of j are all mutations ℓ such that $\ell \prec j$ is a cover relation, where $\ell \prec j$ is a cover relation if no event $j' \in [n]$ exists with $j' \neq \ell, j$ and $\ell \prec j' \prec j$, i.e., cover relations indicate *direct* predecessors. Eq. 1 states that mutation j can occur only after all its predecessor mutations have occurred. Thus, the order relations encoded in the mutation poset $([n], \prec)$ impose constraints on the occurrence of mutations (2).

A mutational pathway is a linear extension of $([n], \prec)$, i.e., a sequence of mutations which is not violating any of the relations. The set of all mutational pathways of length k starting with a minimal element of the poset is denoted $\mathcal{C}_k$ and a typical element is an ordered k-tuple $(j_1, \ldots, j_k)$ of mutations $j_i \in [n]$ with j_1 minimal in the poset and $j_i \prec j_{i+1}$, for all $i = 1, \ldots k-1$. The set $\mathcal{C}_k$ is in one-to-one correspondence with the set of all chains of length $k+1$ in the distributive lattice G of order ideals of $([n], \prec)$ via the mapping $(j_1, \ldots, j_k) \mapsto (\emptyset, \{j_1\}, \{j_1, j_2\}, \ldots, \{j_1, \ldots, j_k\})$. We call G the genotype lattice. It consists of all valid mutation combinations (genotypes) and all possible evolutionary paths connecting them (1).

The probability of a mutational pathway $C \in \mathcal{C}_k$ is given by the product of competing exponentials,

$$\Pr(C = (j_1, \ldots, j_k)) = \prod_{i=1}^{k} \frac{\lambda_i}{\sum_{\ell \in \mathrm{Exit}(j_1, \ldots, j_{i-1})} \lambda_\ell} \tag{2}$$

Here, for any genotype $g \in G$, the exit set $\mathrm{Exit}(g) = \mathrm{Exit}_G(g) = \{j \in [n] \mid j \notin g \text{ and } g \cup \{j\} \in G\}$ consists of all mutations that have not yet occurred in g, but could happen next.

Two examples of mutation posets and their induced genotype lattices are displayed in Fig. S8 and S7. Fig. S8 shows an empty poset, where the n mutations are independent. Consequently, the genotype lattice is the n-dimensional hypercube; all mutational pathways are possible. Neutral passenger mutations are expected to accumulate in this manner. On the other hand, Fig. S7 shows a linear poset. This dependency structure is the strictest among all posets and requires mutations to occur in a single total order. Its genotype lattice consists only of this one mutational pathway. In the following, we will analyze the empty and the linear poset in more detail. They allow for deriving closed-form expressions of the probabilities of interest. The empty poset defines our null model of independence, whereas the linear poset is an example of deviation from the null (dependency structure).

2 The number of preceding mutations

We fix a mutation $m \in [n]$. Let $\mathcal{C}_k(m) = \{(j_1, \ldots, j_k) \in \mathcal{C}_k \mid j_k = m\}$ be the set of mutational pathways ending with mutation m in position k. We are interested in the number of mutations that occur before mutation m. This number is a random variable, because, in general, some mutations will be $\prec$-predecessors of m but some will be (conditionally) independent of m, such that their time of occurrence relative to m is stochastic. This number of preceding mutations of m is

$$A_m = \sum_{\ell=1}^{n} I(T_\ell < T_m) \tag{3}$$

where I is the indicator function. Mutation m has exactly $k-1$ predecessors in a specific realization of the oncogenetic process if the realization is a mutational pathway C that accumulates m as the k-th mutation. Hence, the distribution of A_m is given by

$$\Pr(A_m = k-1) = \sum_{C \in \mathcal{C}_n(k,m)} \Pr(C) \tag{4}$$

where $\mathcal{C}_n(k,m)$ is the set of maximal pathways $(j_1, \ldots, j_n)$ of length n with $j_k = m$. These pathways are defined as all mutation orderings which are compatible with the poset. In the case of the empty poset, every possible order is compatible and the set of all possible orders can be enumerated by permuting the k elements. To compute this probability, it is enough to consider pathways of length k, because

$$\sum_{C \in \mathcal{C}_n(k,m)} \Pr(C) = \sum_{C \in \mathcal{C}_k(m)} \Pr(C) \tag{5}$$

For the empty poset (Fig. S8)

$$1 \quad 2 \quad \ldots \quad m \quad \ldots \quad n$$

$A_m \in \{0, \ldots, n-1\}$. In this case, $\mathcal{C}_k \cong S_k$ is the symmetric group, i.e., the set of all permutations of k mutations, and the cardinality of $\mathcal{C}_n(m)$ is $|\mathcal{C}_n|/n = (n-1)!$. If we assume i.i.d. waiting times, i.e,. the parameters of the exponential waiting time distributions are equal ($\lambda = \lambda_1 = \cdots = \lambda_n$), then, for any maximal path $(j_1, \ldots, j_n)$, the cardinality of the exit set $\mathrm{Exit}(\{j_1, \ldots, j_n\})$ is $n-i$ and

$$\Pr(j_1, \ldots, j_n) = \prod_{i=1}^{n} \frac{\lambda}{(n-i+1)\lambda} = \frac{1}{n!} \tag{6}$$

Thus, as expected for independently and identically occurring mutations, the distribution of the number of mutations preceding m is uniform, $\Pr(A_m = k-1) = (n-1)!/n! = 1/n$, for all $k = 1, \ldots, n$.

For a linear poset (Fig. S7)

$$j_1 \to j_2 \to \cdots \to j_k \to \cdots \to j_n$$

there is only one maximal mutational pathway, $\mathcal{C}_n = \{(j_1, j_2, \ldots, j_n)\}$, and $A_{j_i} = i-1$, hence $\mathcal{C}_k(m) = \emptyset$ unless $j_k = m$, and $\Pr(A_{j_i} = k-1) = 1$ if $i = k$ and zero otherwise.

The cumulative probability of the number of preceding mutations is

$$\Pr(A_m \leq k-1) = \sum_{m=1}^{k} \Pr(A_m = m-1) \tag{7}$$

For the empty poset with identical waiting time distributions, $\Pr(A_m \leq k-1) = \sum_{m-1}^{k} 1/n = k/n$, which is linear in k. For the linear poset, $\Pr(A_{j_i} \leq k-1) = 1$ if $i \leq k$, and 0 otherwise, such that, for fixed $m = j_i$, this probability is a step function in k. This deviating behavior (linear versus step function) is the basis for our mutation timing ranking method. However, since the data is such that the mutation accumulation process is latent and only cross-sectional observations are available, we need to explicitly model the observation (or stopping, or sampling) process and calculate mutation observation probabilities.

3 The number of observed preceding mutations

The mutation accumulation process we have introduced in the above sections only provides a distribution over the order of mutations and their waiting times. However, in real data these orders and waiting times are hidden and we can only observe the mutations statuses at one specific time point, the diagnosis time point.

For the observation process, we model the observation time, or sampling time, as $T_s \sim \text{Exp}(\lambda_s)$ and consider the extended poset $[n] \cup \{s\}$ with no relation between the additional sampling event s and any of the mutations $1, \ldots, n$. The probability of observing the genotype $g \subset [n]$ is the probability of observing all mutation in g and none of its complement $[n] \setminus g$, before s occurred,

$$\Pr(g) = \Pr\left(\max_{j \in g} T_j < T_s < \min_{j \notin g} T_j\right) = \sum_{\{(j_1,\ldots,j_k,s) \in \mathcal{C}_{k+1}(s) \mid \{j_1,\ldots,j_k\} = g\}} \Pr(j_1, \ldots, j_k, s) \tag{8}$$

Let X_m be the binary random variable indicating observation of mutation $m \in [n]$, and denote by $\tau := \sum_{i=1}^{n} X_i$ the total number of observed mutations. We are interested in $\Pr(X_m \mid \tau \leq k)$, the cumulative observation probability of mutation m when a total of k or less mutations have occurred. For the joint probability of X_m and τ, we have

$$\Pr(X_m = 1, \tau = k-1) = \sum_{\{(j_1,\ldots,j_k) \in \mathcal{C}_k(s) \mid m \in \{j_1,\ldots,j_{k-1}\}\}} \Pr(j_1, \ldots, j_k) \tag{9}$$

and

$$\Pr(X_m = 1, \tau \leq k-1) = \sum_{\{(j_1,\ldots,j_k) \in \mathcal{C}_k \mid \exists 1 \leq i \leq k \,:\, j_i = s \,\wedge\, m \in \{j_1,\ldots,j_{i-1}\}\}} \Pr(j_1, \ldots, j_k) \tag{10}$$

Finally, we are interested in the probability

$$P_m(k) = \Pr(X_m = 1 \mid \tau \leq k-1) \tag{11}$$

that among n mutations occurring according to a given CBN model, mutation m has occurred given that a total of $k-1$ or less mutations are observed. This conditional probability will be used for gene ranking.

3.1 Empty poset

Let $\mathcal{E}_k(s,m)$ be the set of all pathways of length k ending in s and containing m, and $\mathcal{E}_k(s,\backslash m)$ the set of all pathways of length k ending in s and not containing m. For the empty poset (Fig. S8), we first compute the joint probability of $X_m = 0$ and $\tau = k-1$,

$$\Pr(X_m = 0, \tau = k-1) = \sum_{C \in \mathcal{C}_k(s,\backslash m)} \prod_{i=1}^{k-1} \frac{\lambda_i}{\sum_{\ell \in \text{Exit}(j_1,\ldots,j_{i-1})} \lambda_\ell} \cdot \frac{\lambda_s}{\sum_{\ell \in \text{Exit}(j_1,\ldots,j_{i-1})} \lambda_\ell} \tag{12}$$

If we again assume identical evolutionary rates, then all pathways of the same length have the same probability. We calculate the cardinalities of the following sets:

$$|\mathcal{C}_k(s)| = \frac{n!}{(n-k)!} \tag{13}$$

$$|\mathcal{E}_k(s,j)| = \frac{(k-1)(n-1)!}{(n-k+1)!} \tag{14}$$

For the probability of a single pathway C, we find

$$\Pr(C) = \prod_{i=1}^{k} \frac{\lambda}{(n-i+2)\lambda} = \frac{(n-k+1)!}{(n+1)!} \tag{15}$$

In order to obtain the joint probability of X_m and τ for identical evolutionary rates, we multiply the probability of a single pathway (Eq. 15) with the cardinality of the set $\mathcal{E}_k(s,m)$ (Eq. 14),

$$\Pr(X_m = 1,\, \tau = k-1) = \frac{(k-1)(n-1)!}{(n-k+1)!} \cdot \frac{(n-k+1)!}{(n+1)!} = \frac{k-1}{n(n+1)} \tag{16}$$

The marginal probability of τ is

$$\Pr(\tau = k-1) = \frac{1}{n+1} \tag{17}$$

and hence we find the conditional probability

$$\Pr(X_m = 1 \mid \tau = k-1) = \frac{k-1}{n(n+1)} \cdot (n+1) = \frac{k-1}{n} \tag{18}$$

The joint probability of $X_m = 1$ and $\tau \leq k-1$ as well as the cumulative marginal probability of τ can be derived by marginalizing over k. We obtain

$$\Pr(X_m = 1,\, \tau \leq k-1) = \sum_{i=1}^{k} \frac{i-1}{n(n+1)} = \frac{k(k+1)-2k}{2n(n+1)} \tag{19}$$

and

$$\Pr(\tau \leq k-1) = \frac{k}{n+1} \tag{20}$$

By multiplying the above joint and marginal probabilities, we finally obtain the conditional probability $P_m(k)$ that is used for gene ranking,

$$P_m(k) = \Pr(X_m = 1 \mid \tau \leq k-1) = \frac{k-1}{2n} \tag{21}$$

For an empty poset, $P_m(k)$ is the probability that among n independent mutations a specific mutation has occured when $k-1$ or less mutations are observed in total. It is a linear function in k.

3.2 Linear poset

For ease of notation we set $\lambda_{j_{n+1}} = 0$. For the linear poset (Fig. S7), we find

$$\Pr(X_m = 0,\, \tau = k-1) = \Pr(j_1, \ldots, j_{k-1}, s) = \prod_{i=1}^{k-1} \frac{\lambda_{j_i}}{\lambda_{j_i} + \lambda_s} \cdot \frac{\lambda_s}{\lambda_{j_k} + \lambda_s} \tag{22}$$

if $m \notin \{j_1, \ldots, j_{k-1}\}$, and zero otherwise. For identical evolutionary rates, $\lambda_1 = \ldots = \lambda_n = \lambda_s$, this probability becomes 2^{-k}. The marginal probability of observing $k-1$ mutations is

$$\Pr(\tau = k-1) = \Pr(j_1, \ldots, j_{k-1}, s) \tag{23}$$

For the conditional probability, we find the step function

$$\Pr(X_m = 0 \mid \tau = k-1) = \frac{\Pr(j_1, \ldots, j_{k-1}, s)}{\Pr(j_1, \ldots, j_{k-1}, s)} = 1 \tag{24}$$

if $m \notin \{j_1, \ldots, j_{k-1}\}$, and zero otherwise.

Let p be the position of m in the poset, i.e., $m = j_p$. The cumulative marginal probability of τ is

$$\Pr(\tau \leq k-1) = \sum_{i=1}^{k-1} \prod_{l=1}^{i} \frac{\lambda_{j_i}}{\lambda_{j_i} + \lambda_s} \cdot \frac{\lambda_s}{\lambda_{j_k} + \lambda_s} \tag{25}$$

and for the conditional probability, we find

$$\Pr(X_m = 0 \mid \tau \leq k-1) = \left(\sum_{i=1}^{k-1} I(i<p) \prod_{l=1}^{i} \frac{\lambda_{j_i}}{\lambda_{j_i}+\lambda_s} \cdot \frac{\lambda_s}{\lambda_{j_k}+\lambda_s} \right) \cdot \left(\sum_{i=1}^{k-1} \prod_{l=1}^{i} \frac{\lambda_{j_i}}{\lambda_{j_i}+\lambda_s} \cdot \frac{\lambda_s}{\lambda_{j_k}+\lambda_s} \right)^{-1} \tag{26}$$

for $p < k$, and zero otherwise. Assuming identical evolutionary rates, this probability simplifies to

$$\Pr(X_m = 0 \mid \tau \leq k-1) = \sum_{l=1}^{p} 2^{-l} \cdot \left(\sum_{l=1}^{k} 2^{-l} \right)^{-1} = \frac{1-2^{-p}}{1-2^{-k}} \tag{27}$$

Similarly,

$$\Pr(X_m = 1, \tau \leq k-1) = \left(\sum_{i=1}^{k-1} I(i \geq p) \prod_{l=1}^{i} \frac{\lambda_{j_i}}{\lambda_{j_i}+\lambda_s} \cdot \frac{\lambda_s}{\lambda_{j_k}+\lambda_s} \right) \tag{28}$$

and for identical evoluionary rates,

$$\Pr(X_m = 1, \tau \leq k-1) = \sum_{l=1}^{k} \left(\frac{1}{2}\right)^{l} - \sum_{l=1}^{p} \left(\frac{1}{2}\right)^{l} = \frac{1}{2^p} - \frac{1}{2^k} \tag{29}$$

for $p < k$, and one otherwise. Hence, for linear posets, the conditional probability of interest, $P_m(k)$, that is used for ranking genes is

$$P_m(k) = \Pr(X_m = 1 \mid \tau \leq k-1) = \begin{cases} \frac{2^{-p}-2^{-k}}{1-2^{-k}} & \text{if } p < k \\ 0 & \text{otherwise} \end{cases} \tag{30}$$

This probability, as a function of k, is zero until $k = p$, then rises sharply, and then levels off (Fig. S9). In the linear poset case, $P_m(k)$ is the probability of observing the p-th mutation in a linear chain when a total of $k-1$ or less mutations are observed.

4 The steepest slope of $P_m(k)$

For gene ranking, we consider the conditional probability $P_m(k) = \Pr(X_m = 1 \mid \tau \leq k-1)$ (Eq. 11) for different posets. We continue to assume identical rates of evolution for all mutations in this section, $\lambda = \lambda_1 = \cdots = \lambda_n$. We show that after rescaling, for any poset with a relation involving m, $P_m(k)$ has a higher maximal slope than for the empty poset, which has constant slope (Eq. 21). In other words, mutational dependencies on or of m can be detected by considering the steepest slope of $P_m(k)$.

In order to compare highest slopes among posets, we first rescale $P_m(k)$ to be 1 at $k = n$, and define the mutation accumulation function

$$Q_m(k) = \frac{P_m(k)}{P_m(n)} \tag{31}$$

such that its largest value, i.e., the overall marginal frequency of mutation m, is $Q_m(n) = 1$.

For n independent mutations (empty poset), $P_m^{\text{ind}}(k) = (k-1)/(2n)$ is linear in k, with constant slope $1/(2n)$ (Eq. 21) and marginal mutation frequency $P_m^{\text{ind}}(n) = (n-1)/(2n)$ for any mutation $m \in [n]$. Hence,

$$Q_m^{\text{ind}}(k) = \frac{2n}{n-1} \frac{k-1}{2n} = \frac{k-1}{n-1} \tag{32}$$

has constant slope $1/(n-1)$.

We first compare this slope to the steepest slope of Q for a linear poset. If the mutations are totally ordered, and mutation $m = j_p \in [n]$ occurs at position p of the linear chain, then $P_m^{\text{lin}}(k)$ is zero for $k < p$ and for $k \geq p$, it increases in a non-linear fashion as $(2^{-p} - 2^{-k})/(1-2^{-k})$ (Eq. 30). The marginal frequency of mutation m is $(2^{-p} - 2^{-n})/(1-2^{-n})$. Hence,

$$Q_m^{\text{lin}}(k) = \frac{1-2^{-n}}{2^{-p}-2^{-n}} \frac{2^{-p}-2^{-k}}{1-2^{-k}} \tag{33}$$

if $k \geq p$, and zero otherwise. The highest slope of Q_m^{lin} is at $k = p$, namely

$$\frac{\partial Q_m^{\text{lin}}(k)}{\partial k}(p) = \frac{1-2^{-n}}{2^{-p}-2^{-n}} \frac{\log(2)}{2^p - 1} \tag{34}$$

When comparing the highest slopes between the empty and the linear poset, we find that

$$\frac{\partial Q_m^{\text{ind}}(k)}{\partial k}(m) < \frac{\partial Q_m^{\text{lin}}(k)}{\partial k}(p) \tag{35}$$

for all m, if and only if

$$\frac{1}{n-1} < \frac{1-2^{-n}}{2^{-p}-2^{-n}} \cdot \frac{\log(2)}{2^p - 1} \tag{36}$$

This inequality is fulfilled for all $1 \leq p \leq n$. Thus, the mutation accumulation function Q increases much steeper for totally ordered mutations than for independent mutations.

Next, we relax the condition of total mutation order and generalize this observation to the situation where a mutation m has only at least one upstream or downstream dependency. For any given poset with this property, we denote by $P_m^{\text{dep}}(k)$ and $Q_m^{\text{dep}}(k)$ the corresponding conditional and scaled conditional probability, respectively.

Theorem 1. Consider a mutation m in a CBN model with identical evolutionary rates. If the poset contains either a relation $m' \prec m$ for a mutation $m' \neq m$ or a relation $m \prec m''$ for a mutation $m'' \neq m$ and for all of these relations, m'' has no further upstream dependencies, then there is an index ℓ such that

$$\frac{\partial Q_m^{\text{dep}}(k)}{\partial k}(\ell) > \frac{\partial Q_m^{\text{ind}}(k)}{\partial k}(\ell)$$

that is, such that its scaled mutation accumulation function has steepest slope larger than in the case of independent mutations occurring at the same evolutionary rate.

Proof. We proceed by considering the two alternative (but non-exclusive) assumptions separately. In the first case, m is dependent on at least one event (i.e., has an upstream dependencies). The mutation accumulation function $Q_m^{\text{dep}}(k)$ is monotonically increasing in k, because mutations are not reversible and therefore the (scaled) conditional probability can only stay constant or increase with k. Furthermore, $Q_m^{\text{dep}}(k)$ is 0 at least until $k = 2$. This is because m can not occur before all its upstream dependent events have occurred. For the empty poset, $Q_m^{\text{ind}}(k)$ can be written as $(k-1) \cdot h$ where $h^{\text{ind}} = 1/(n-1)$ is the constant step size for the increase of the accumulation function (Eq. 32). Furthermore, $Q_m^{\text{dep}}(k)$ can be written as $\sum_{t=1}^{k} h_t$ where h_t is the increase of the accumulation function at time t. We know that $h_1 = 0$ and $h_t \geq 0$ for all t. Hence, there is an index $\ell \in \{2, \ldots, n\}$ such that $h_\ell > h^{\text{ind}}$, i.e., the largest increase of $Q_m^{\text{dep}}(k)$ is higher than that of $Q_m^{\text{ind}}(k)$.

Let us assume now that mutation m has only downstream dependencies. We first analyze the situation where m has $n-2$ direct downstream dependencies and no further dependencies. If $P_m^{\text{dep}}(k)$ was a linear function in k, then it would hold that $P_m^{\text{dep}}(3) - P_m^{\text{dep}}(2) = P_m^{\text{dep}}(2) - P_m^{\text{dep}}(1)$. Since m has only direct downstream dependencies, we can explicitly calculate the three conditional probabilities. First,

$$P_m^{\text{dep}}(1) = 0$$

because if only one event is observed, then it could only have been the diagnosis event. Next,

$$P_m^{\text{dep}}(2) = \left[\frac{\lambda}{2\lambda} \cdot \frac{\lambda}{(n-1)\lambda}\right] \Big/ \left[\frac{\lambda}{2\lambda} + \frac{\lambda}{2\lambda} \cdot \frac{\lambda}{(n-1)\lambda}\right] = \frac{1}{n}$$

where the numerator is the probability that mutation m occurred right before the diagnosis event, and the denominator is the probability that two or less events happened. Similarly,

$$\begin{aligned} P_m^{\text{dep}}(3) &= \left[\frac{\lambda}{2\lambda} \cdot \frac{\lambda}{(n-1)\lambda} + \frac{\lambda}{2\lambda} \cdot \frac{(n-2)\lambda}{(n-1)\lambda} \cdot \frac{\lambda}{(n-2)\lambda}\right] \Big/ \left[\frac{\lambda}{2\lambda} + \frac{\lambda}{2\lambda} \cdot \frac{\lambda}{(n-1)\lambda} + \frac{\lambda}{2\lambda} \cdot \frac{(n-2)\lambda}{(n-1)\lambda} \cdot \frac{\lambda}{(n-2)\lambda}\right] \\ &= \frac{2}{n+1} \end{aligned}$$

follows the same structure as $P_m^{\text{dep}}(2)$. With these expression, the above relation yields, after simplifying, $n = n + 1$, a contradiction. Thus, $P_m^{\text{dep}}(k)$ is not linear and there must be a point between $k = 1$ and $k = n$ where the slope of $Q_m^{\text{dep}}(k)$ is larger then the one of Q_m^{ind}.

Finally, let us assume that mutation m has downstream dependencies that have further downstream dependencies. Following the case above, we know that $Q_m^{\text{dep}}(2) = (1/n)/P_m(n)$. This is because, as compared to the situation above, only additional downstream nodes are added and this does not influence $P_m^{\text{dep}}(2)$. For the empty poset, we have $Q_m^{\text{ind}}(2) = 1/(n-1)$, such that $Q_m^{\text{dep}}(2) > Q_m^{\text{ind}}(2)$ if and only if $(n-1)/n > P_m^{\text{dep}}(n)$, which is true for all $n > 2$, because $P_m^{\text{dep}}(n)$ is bounded from above by $P_m^{\text{ind}}(n) = (n-1)/(2n) < 1/2$. Thus, at $k = 2$, we find a steeper slope than for the empty poset. This concludes the proof. □

4.1 Approximating with sigmoid functions and ranking by slope

In practice, we want a robust estimate of the steepest slope of $Q_m(k)$. For this purpose, we approximate $Q_m(k)$ by a sigmoid function $f_m(k)$, and the steepest slope (Eq. 34) is an upper bound for the expected slope of the sigmoidal curve. To obtain a better estimate, we consider the average slope of $Q_m(k)$ after the initial increase. Specifically, we analyze at which point this probability crosses the 90% threshold. For large n, $Q_m(n) \approx 2^{-p}$. Thus, we consider

$$\frac{2^{-p} - 2^{-k}}{1 - 2^{-k}} = 0.9 \cdot 2^{-p} \tag{37}$$

This equation has the solution

$$k = \log_2(10(-0.9 + 2^p)) \tag{38}$$

which can be approximated by $k = p+3$ (Fig. S10), i.e., after $k > p+3$ the conditional probability $P_m^{\text{linear}}(k)$ is larger than 90% of its asymptote. If we linearly interpolate the rise from 0 at $k = p$ to the 90% of the asymptote at $k = p + 3$, then we find an average slope of $0.9/3 = 0.3$ for the linear poset. This compares to the (scaled) exact value of $\log(2)/(1 - 2^{-p})$, or approximately $\log(2) \approx 0.69$ for large p.

For the empty poset, 90% of its increase will be achieved after k passed 90% of $n + 1$ which is the maximum value for k. Thus, the (scaled) average slope is $0.9/n$. Comparing the independent and linear cases, we find a steeper average slope in the linear case, if and only if $0.3 > 0.9/n$ or $n > 3$.

References

[1] N. Beerenwinkel, N. Eriksson, and B. Sturmfels. Conjunctive Bayesian networks. *Bernoulli*, 13(4):893–909, November 2007.

[2] N. Beerenwinkel and S. Sullivant. Markov models for accumulating mutations. *Biometrika*, 96(3):645–661, June 2009.

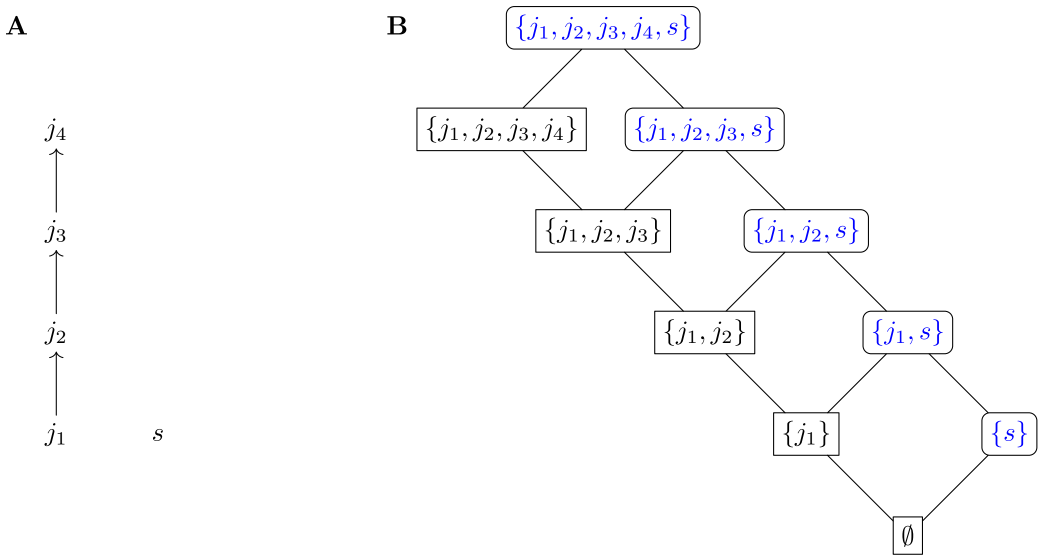

Figure S7: Linear order constraints among $n = 4$ mutations. (A) Linear poset of four events and independent sampling event s. (B) The induced genotype lattice for the linear poset shown in panel A. The nodes are valid mutational states (i.e., genotypes compatible with the order constraints). Transitions can only occur upwards along the edges. Observable states are shown in blue font inside rounded boxes.

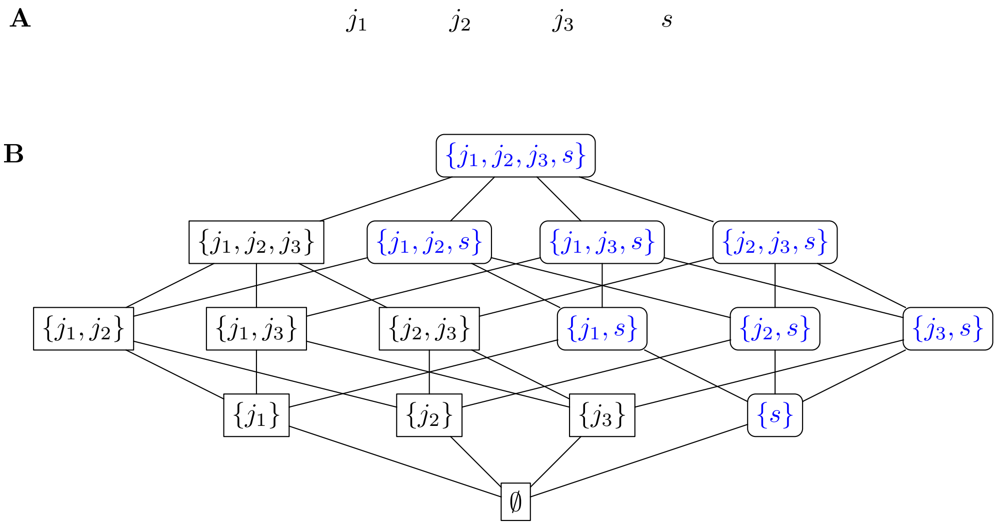

Figure S8: Three independent mutations ($n = 3$). (A) Empty poset (no order constraints) of three events and independent sampling event s. (B) The induced genotype lattice for the empty poset shown in panel A consists of all possible combinations of mutations (i.e., all genotypes are valid). Transitions can only occur upwards along the edges. Observable states are shown in blue font inside rounded boxes.

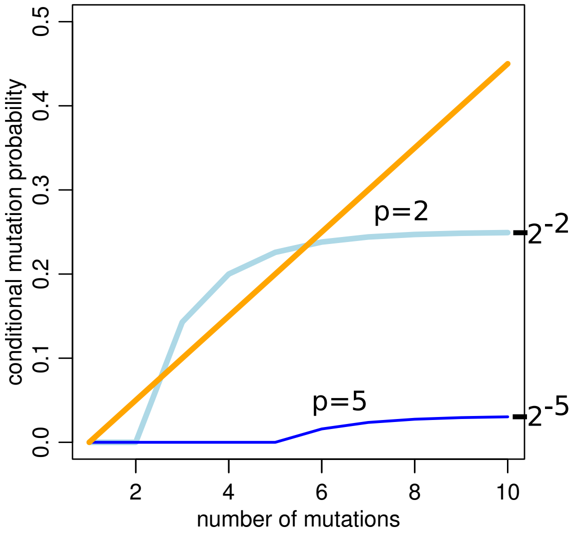

Figure S9: Conditional mutation probabilities for the linear and the empty poset. The curves depict the conditional probability $P_m(k) = \Pr(X_m = 1 \mid \tau \leq k-1)$ of gene m being mutated given that less than k mutations have occurred. For the linear poset, mutation $m = j_p$ at position $p = 2$ (light blue) and $p = 5$ (dark blue) of the linear chain are shown. The asymptote of $P_m(k)$, i.e., the overall marginal frequency of mutation m, which is 2^{-p} for the linear poset, is indicated to the right. For independent mutations, $P_m(k)$ is shown in orange.

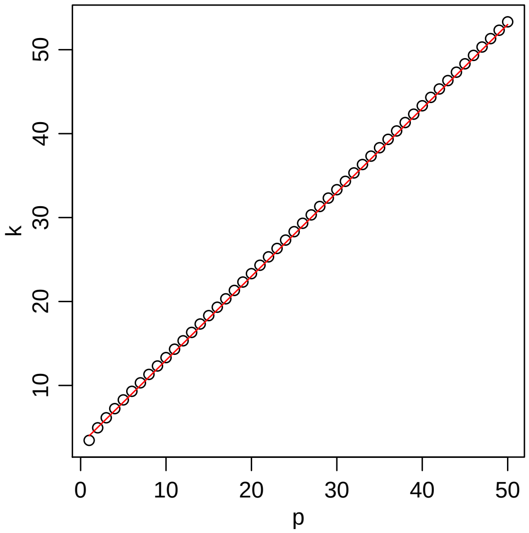

Figure S10: Approximate slope for the linear poset. Shown is the number of additional mutations (to p) needed to reach 90% of the cumulative limit probability for a linear poset. Circles denote $k = \log_2(10(-0.9 + 2^p))$ and the red line is $k = p + 3$.
